# Outdoor walking exhibits peak ankle and knee flexion differences compared to fixed and adaptive-speed treadmills in older adults

**DOI:** 10.1186/s12938-021-00941-0

**Published:** 2021-10-15

**Authors:** Sheridan M. Parker, Jeremy Crenshaw, Nathaniel H. Hunt, Christopher Burcal, Brian A. Knarr

**Affiliations:** 1 Department of Biomechanics, University of Nebraska at Omaha, 6160 University Dr S., Omaha, NE 68182 USA; 2 Department of Kinesiology and Applied Physiology, University of Delaware, Newark, DE USA; 3 School of Health and Kinesiology, University of Nebraska at Omaha, Omaha, NE USA

**Keywords:** Adaptive-speed treadmill, Walking speed, Kinematics, Joint angles, Older adults

## Abstract

**Background:**

Walking mechanics recorded with a traditional treadmill may not be the same as the mechanics exhibited during activities of daily living due to constrained walking speeds. Adaptive-speed treadmills allow for unconstrained walking speeds similar to outdoor walking. The aim of this study was to determine differences in kinematic walking parameters of older adults between adaptive-speed treadmill (AST), fixed-speed treadmill (FST) and outdoor walking. We hypothesized that self-selected walking speed (SSWS) during AST walking and outdoor walking would increase compared to FST walking. Furthermore, we hypothesized that AST walking and outdoor walking would increase peak knee flexion, hip flexion, and ankle plantarflexion angles compared to FST walking independent of walking speed changes.

**Methods:**

Fourteen older adult participants were asked to complete 3 min of FST and AST walking on a split-belt treadmill. Participants were also asked to complete 6 min of outdoor walking following a circular route in a neighboring park. A wireless inertial measurement unit-based motion capture system was used to record lower extremity kinematics during all walking conditions.

**Results:**

The outdoor walking condition produces significantly higher SSWS compared to FST (*p* < 0.001) and AST (*p* = 0.02) conditions. A significantly faster SSWS was exhibited during the AST condition compared to the FST condition (*p* = 0.026). Significantly higher peak ankle plantarflexion angles are exhibited during the outdoor walking condition compared to the AST (*p* < 0.001, *g* = 1.14) and FST (*p* < 0.001, *g* = 1.13) conditions after accounting for walking speed. There was a significantly lowered difference between the outdoor walking condition and both AST (*p* = 0.029, *g* = 0.49) and FST (*p* = 0.013, *g* = 0.63) conditions in peak knee flexion angles after accounting for SSWS. There are no significant differences between outdoor, AST, and FST conditions on peak hip flexion angles. Older adults exhibit changes in peak ankle plantarflexion and peak knee flexion angles during outdoor walking compared to treadmill walking but not between treadmill controller types. We found no differences in the kinematics exhibited by older adults between both AST and FST walking.

**Conclusions:**

Incorporating unconstrained walking speed with the AST while maintaining similar FST sagittal plane kinematics may allow for more translatable conditional balance and walking rehabilitation.

## Background

Treadmill walking allows for increased continuous motion and requires less laboratory space compared to overground walking, where spatial constraints often limit the amount of continuous walking that can be performed [1]. However, previous literature has determined that treadmill walking may change the kinematics of individuals compared to overground walking [2–4]. Dingwell et al. [2] found that treadmill walking is associated with overestimates of stability through lowered kinematic variability, compared to overground walking and therefore argues that treadmills should not be used in specific situations. These potential situations could include research on falls or with participant populations that typically experience instability. A study by Hollman et al. [3] corroborates these findings and further argues that walking mechanics developed through treadmill training may not be transferable to overground walking. Treadmills also require additional tasks beyond walking propulsion, balance, and body weight support. Traditional treadmills require that an individual’s walking speed be controlled. Step length and step time may vary while walking on a traditional treadmill; the combination of these two, forward walking speed, needs to be controlled so that the individual does not walk off the front or fall off the back of the treadmill [5]. These findings suggest that walking mechanics recorded with traditional treadmill walking might not be the same as the mechanics exhibited during activities of daily living.

Previous literature has primarily investigated walking stability in the context of optimal laboratory conditions with the use of force plates and standardized treadmills [2, 3, 6–9]. While these studies provide critical insight into walking stability, the risk of falls and walking mechanics experienced by an older adult population might not be the same in an optimal environment as compared to realistic environments experienced during activities of daily living. A study by Rispens et al. [10] compared trunk acceleration as a measure of stability between treadmill walking and a community-dwelling environment. They found that walking in a community-dwelling environment resulted in less stability than when walking on a treadmill [10]. To fully understand the relationship between walking mechanics and risk of falls, they must be studied in the context of daily living. However, community-dwelling environments have many uncontrollable variables and the use of traditional research equipment, such as force plates and motion capture cameras, are restricted compared to a traditional laboratory setting. As an alternative, inducing more ecologically valid walking mechanics within an optimal laboratory environment may aid in providing an accurate understanding of walking mechanics and their contribution to fall risk.

Previous studies have used an Adaptive Speed Treadmill (AST) controller to emulate community-dwelling walking by incorporating unconstrained walking speed as a factor into the novel treadmill controller [11, 12]. Using this custom AST controller walking both of the split-belt treadmill belts, change speed simultaneously in response to the impulse of the instantaneous anterior inertial force, step length and duration, and the position of the individual relative to the center of the treadmill [12]. Calculations for this AST controller are made concurrently for both limbs with respect to foot placement on the treadmill and walking phase. A study by Ray et al. [12] determined that the AST induces a more natural walking experience in healthy young adults by allowing the AST to adapt to the user’s specific walking speed variability [12]; whereas, with a fixed-speed treadmill (FST), the user would have to constrain their walking speed to the treadmill’s constant speed. Previous literature focused on young healthy participants reports that the AST induces kinematic ankle, knee and hip variability, using the Lyapunov exponent that is comparable to FST walking [13]. Many studies that have used an AST have focused on young healthy adults and individuals with stroke [12–14], however, the effectiveness of the AST to induce a more natural walking experience and the effect of AST walking on older adult stability is not well understood within the older adult population. By focusing on walking mechanics, we further our understanding of the use of the AST and the extent to which the AST can be used within a laboratory environment for the older adult population.

### Aim

The aim of this study was to determine differences in kinematic walking parameters of older adults between AST, FST, and outdoor walking. Specifically, we focused on peak ankle plantarflexion, peak knee flexion, and peak hip flexion angles, as these are common measures of walking mechanics that influence walking speed and thus quality of life [15, 16].

### Hypotheses

We hypothesized that older adult self-selected walking speeds (SSWS) during AST walking and outdoor walking would increase compared to FST walking, as treadmill controller type would affect older adult SSWS due to the incorporation of unconstrained walking speed. Literature has reported decreased gait speed in daily living compared to laboratory conditions however, the collection of walking measures over multiple days could affect walking speed due to differences in walking durations [17–19]. The inclusion of unconstrained walking speeds in the AST controller will allow for participants to self-select their walking speeds similar to outdoor walking that resembles overground walking. Previous literature has reported similar outcomes in young adults where the AST condition exhibited faster self-selected walking speeds compared to the FST condition but no difference with overground walking speeds [12].

We also hypothesized that AST walking and outdoor walking would increase peak knee flexion angles, increase peak hip flexion angles, and increase ankle plantarflexion angles compared to FST walking as the AST controller incorporates unconstrained walking speed. The inclusion of unconstrained walking speed in this study will allow for participants to self-select their comfortable walking speed which will also allow for unbiased kinematic joint angles which change with modulated walking speeds [4, 6, 12].

Furthermore, we hypothesize that the differences in kinematics observed between conditions will be independent of walking speed changes. While walking speed can influence kinematics, the observed differences in the outcomes will result from differences in walking conditions.

## Results

### Self-selected walking speeds

**Fig. 1:**
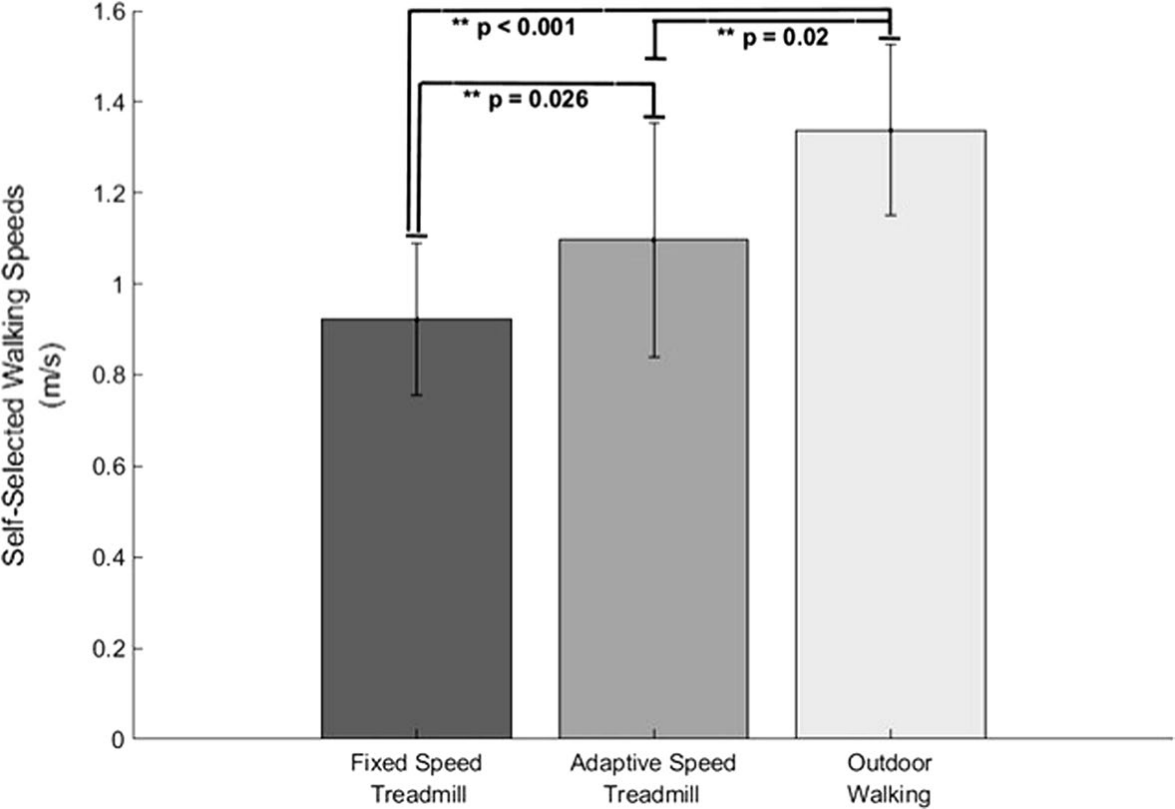
Mean SSWS results. Error bars represent standard deviation. The outdoor walking condition produces significantly higher SSWS compared to FST (*p* < 0.001) and AST (*p* = 0.02) conditions. A significantly faster SSWS was exhibited during the AST condition compared to the FST condition (*p* = 0.026)

A repeated-measures ANOVA indicated that there was a significant difference in SSWS between walking conditions (*p* < 0.001, η^2^_*p*_ = 0.627). Participants walked significantly faster outdoors than during the FST (*p* < 0.001, *g* = 0.51) and AST (*p* = 0.02, *g* = 0.64) walking conditions (Fig. 1). There was also a significant difference between AST and FST SSWSs (*p* = 0.026, *g* = 0.62).

### Peak ankle plantar flexion

When not accounting for walking speed, there was a statistically significant difference in peak ankle plantarflexion between the walking conditions (*p* < 0.001, *η*^2^_*p*_ = 0.577). A significant increase in peak ankle plantarflexion occurred during the outdoor condition compared to the AST (*p* = 0.001, *g* = 1.14) and FST (*p* < 0.001, *g* = 1.13) walking conditions (Table 1). There was no significant difference between the AST and FST walking conditions (*p* = 0.463, *g* = 0.18) on peak ankle plantarflexion angles.

**Table 1:** Peak joint angles (*M* ± SD, degrees) exhibited during each walking condition

Peak joint angle	Adaptive-speed treadmill	Fixed-speed treadmill	Outdoor walking
Ankle plantarflexion (*n* = 14)	16.4 ± 9.5	15.5 ± 9.6	24.2 ± 8.4
Knee flexion (*n* = 14)	59.1 ± 4.7	59.0 ± 5.0	56.6 ± 4.7
Hip flexion (*n* = 14)	16.8 ± 5.2	15.4 ± 4.3	17.6 ± 5.7

**Fig. 2:**
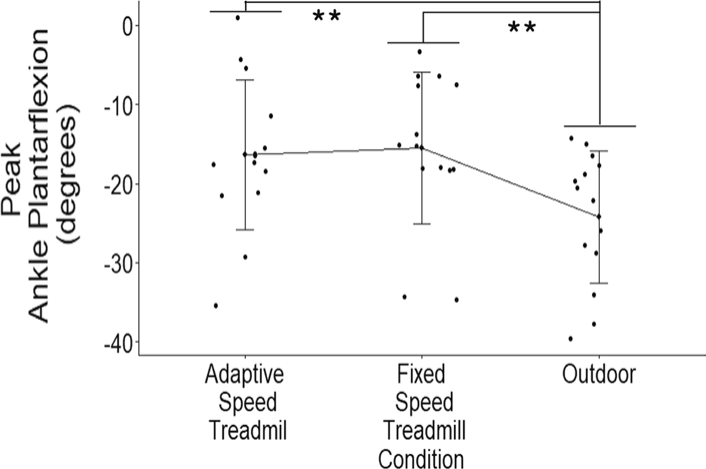
Individual participant results by walking condition with error bars representing standard deviation. ** indicates statistical significance. Significantly higher peak ankle plantarflexion angles are exhibited during the outdoor walking condition compared to the AST (*p* < 0.001, *g* = 1.14) and FST (*p* < 0.001, *g* = 1.13) conditions after accounting for walking speed

The repeated-measures ANCOVA indicated that there was a statistically significant difference between walking conditions (*p* = 0.016, *η*^2^_*p*_=0.340). A large effect indicating a significant increase in peak ankle plantarflexion occurred during the outdoor condition compared to both the AST (*p* < 0.001, *g* = 1.14) and FST (*p* < 0.001, *g* = 1.13) conditions after accounting for walking speed (Fig. 2). There was no significant difference in peak ankle plantarflexion between the AST and FST conditions (*p* = 0.303, *g* = 0.18) with a small effect size after accounting for walking speed. Means (M) and standard deviations (SD) for peak joint angles exhibited during each walking condition can be seen in Table 1.

### Peak knee flexion

When not accounting for walking speed, there was a statistically significant difference between walking conditions on peak knee flexion angles (*p* = 0.023, *η*^2^_*p*_ = 0.253). There was no significant difference between the AST and outdoor walking conditions on peak knee flexion angles (*p* = 0.063, *g* = 0.49). There was a significant decrease in exhibited peak knee flexion angles during the outdoor condition compared to the FST condition (*p* = 0.021, *g* = 0.63). There is no significant difference between the AST and FST conditions (*p* = 0.871, *g* = 0.04) on peak knee flexion angles when not accounting for walking speed.

**Fig. 3:**
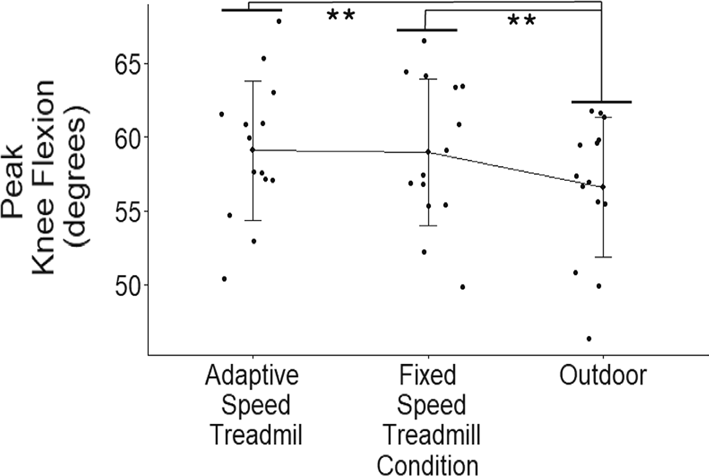
Individual participant results by walking condition with error bars representing standard deviation. ** indicates statistical difference. There was a significantly lowered difference between the outdoor walking condition and both AST (*p* = 0.029, *g* = 0.49) and FST (*p* = 0.013, *g* = 0.63) conditions in peak knee flexion angles after accounting for SSWS

The repeated-measures ANCOVA indicated that there was a statistical difference between AST, FST, and outdoor walking conditions on peak knee flexion angles (*p* = 0.01, *η*^2^_*p*_ = 0.367, Table 1). A decrease in exhibited peak knee flexion angle during the outdoor walking condition compared to the AST walking condition was determined resulting in a small statistically significant effect after accounting for walking speed (*p* = 0.029, *g* = 0.49). Peak knee flexion angles were also decreased during the outdoor condition compared to the FST walking condition resulting in a medium statistically significant effect after accounting for walking speed (*p* = 0.013, *g* = 0.63) (Fig. 3). There was a small non-significant difference between the AST and FST conditions on peak knee flexion angles (*p* = 0.856, *g* = 0.04).

### Peak hip flexion

**Fig. 4:**
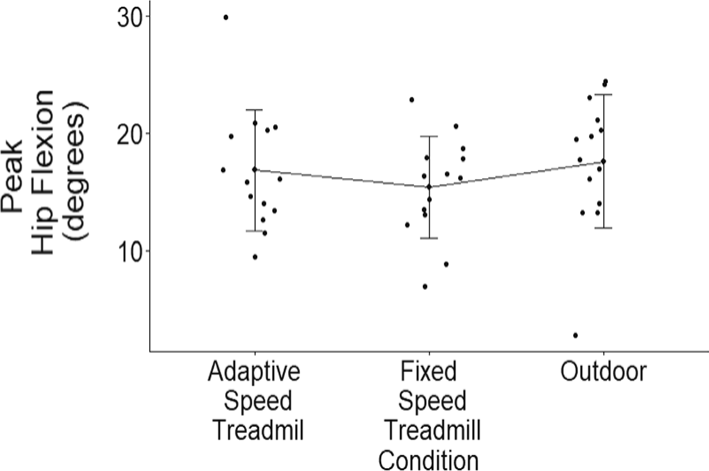
Individual participant results grouped by walking condition with error bars representing standard deviation. There are no significant differences between outdoor, AST, and FST conditions on peak hip flexion angles

The Friedman’s test indicated that there was no statistically significant difference between outdoor, AST, and FST walking conditions (*Χ*^2^ = 1.86, *p* = 0.395) on peak hip flexion angles after accounting for walking speed (Table 1, Fig. 4).

## Discussion

The aim of this study was to determine differences in kinematic walking parameters of older adults between AST, FST, and outdoor walking. We investigated peak ankle plantarflexion peak knee flexion, and peak hip flexion as measures to understand how walking mechanics change in response to different walking tasks. Kinematics are also associated with kinetics, which are common measures of walking mechanics thought to influence walking stability and quality of walking [15, 16]. We also compared the kinematic measures exhibited during the AST and FST walking conditions to the kinematic measures exhibited during outdoor walking. This comparison to outdoor walking assists in understanding the ecological validity of how kinematic changes occur between walking in an outdoor community-dwelling environment compared to treadmill walking.

### Self-selected walking speeds

Older adults walked at significantly faster SSWSs outdoors compared to AST and FST walking conditions. Therefore, our first hypothesis is partially supported. In a previous study, Ray et al. [12] reported that the AST condition produced faster SSWSs than the FST condition in young adults. The results from our study agree with this finding in that there is a statistically significant increase in walking speed during the AST walking condition. The SSWSs recorded during the AST condition are closer to the outdoor walking condition than to the FST walking condition. The inclusion of unconstrained walking speed as a factor allows the AST to emulate outdoor walking speeds more closely. These results mirror Ray et al. [12] where young healthy participants increased their SSWSs on an AST to similar speeds as overground walking. The benefits of an AST are that it allows individuals to fluctuate their walking speeds as they would during outdoor walking which may allow for a more translatable condition in balance and walking rehabilitation. This allows clinicians to be able to monitor and provide rehabilitation to individuals with balance or walking impairments in a controlled environment while individuals are able to fluctuate their walking speeds as if they were outdoors walking.

### Peak ankle plantar flexion

Older adults exhibited increased peak ankle plantarflexion angles during the outdoor walking condition compared to the AST and FST conditions when controlling for SSWSs. Therefore, our second hypothesis is partially supported. A major component to the AST controller is the instantaneous anterior inertial force, also called the push-off force. This push-off force is often modulated through changes in ankle plantarflexion late in the stance phase. A study by Barak et al. [6] reported a main effect of speed on peak ankle plantarflexion. Specifically, peak ankle plantarflexion increased with increased walking speed. Our results agree with the previous literature in that the outdoor walking condition exhibited increased walking speeds compared to the treadmill conditions along with increased peak ankle plantarflexion angles. This could mean that older adults are increasing their peak plantarflexion angle and thus increasing their push-off force to increase their walking speeds while walking outdoors compared to treadmill walking. When we did not control for SSWSs, the same results occurred. This indicates that the differences in the peak ankle plantarflexion angles are independent of walking speeds and our third hypothesis is supported.

Specifically looking at walking condition comparisons, there is no consensus on how changes in walking condition influence peak ankle plantarflexion. A study by Sloot et al. [4] used a different type of adaptive-speed treadmill controller that also allows for unconstrained walking speeds and found that FST walking resulted in a 0.6° increase in peak ankle plantarflexion angles from AST walking. This small increase in peak ankle plantarflexion angle is not a meaningful difference, so it can be inferred that they found no significant difference between AST and FST walking conditions [4]. Therefore, our results support previous literature in that we also found no difference in peak ankle plantarflexion angles between AST and FST walking [4, 20]. We also found an increase in peak ankle plantarflexion angles during outdoor walking compared to treadmill walking. This indicates that the usability of the AST is just as good as FST although may still be limited based on being a treadmill controller. Even though the AST controller changes the treadmill belt speed for each step there still may be a contribution from the treadmill belts to pull the users’ limb posterior during the stance phase. Whereas with outdoor walking the individuals’ limb would not be pulled posterior by the ground therefore the individual may have to contribute more plantarflexion during the push-off phase of walking. When walking speed was controlled, the differences we observed in peak ankle plantarflexion angles were the result of differences in walking conditions directly. Peak ankle plantarflexion was affected by changes in walking speed where we saw that participants walked faster outdoors and, on the AST, compared to FST walking (Fig. 1). When walking speed was not a covariate in the analysis, the results indicated no changes in the peak ankle plantarflexion results compared to when walking speed is accounted for. This indicates that the changes in walking speed between walking conditions did not modulate peak ankle plantarflexion angles within this study.

### Peak knee flexion

There is a significant difference between AST, FST, and Outdoor walking conditions on peak knee flexion angles when controlling for self-selected waking speeds. Specifically decreased peak knee flexion angles are exhibited during the outdoor condition compared both treadmill conditions while there was no significant difference between AST and FST conditions. Therefore, our second hypothesis is not supported. The decrease in peak knee flexion angle during outdoor walking could indicate a change in lower limb clearance during swing. Our results corroborate Sloot et al. [4] who also found no difference between FST walking and a different type of AST controller walking in peak knee flexion angles. The difference seen between outdoor walking and treadmill walking conditions could be due to difference in walking speed where participants chose to walk with a faster SSWS while outdoors.

When walking speed is not accounted for in peak knee flexion angle analysis, a significant difference between the outdoor and AST conditions arises that is not present when walking speed is accounted for. The changes in walking speed between walking conditions influences the exhibited peak knee flexion angles even though this same effect is not observed in peak ankle plantarflexion angles within this study. This could indicate that participants are self-selecting slower walking speeds while walking on treadmills, both fixed and adaptive-speed, which influences their walking mechanics [12–14, 21]. Therefore, our third hypothesis is not supported as the main effects seen in peak knee flexion angles are not independent of SSWSs.

### Peak hip flexion

There is no statistical difference in peak hip flexion angles between AST, FST, and outdoor walking conditions. Even though there are significant differences in both peak ankle plantarflexion angles and peak knee flexion angles between walking condition, the main effect is not exhibited in peak hip flexion angles. Therefore, our second hypothesis is not supported. This indicates that participants are potentially exhibiting more contribution from changes in ankle and knee joint angles to modulate walking mechanics between conditions or through other means. Previous literature has reported that peak hip flexion angles significantly increase with increasing walking speeds [6]. The exhibited peak hip flexion angles collected in our study also increase during conditions with increased SSWS. However, we did not see a statistical difference in peak hip flexion angles between walking conditions that have increased SSWS. Therefore, we can conclude that our third hypothesis is supported.

## Limitations

The first limitation of this study is that we focused our analysis specifically on kinematics as we do not have kinetic data due to using the inertial motion capture system outdoors. While peak joint angle analyses provided insight into kinematic changes between the walking conditions, there may be more significant differences that occur when focusing comprehensively on stride cycle-based analyses by incorporating kinetics. The acclimation period may be a limitation as well, we implemented a 2-min acclimation period, however there may be increased differences between the AST and FST conditions if the acclimation period was longer, or an accommodation program was implemented to allow the participants more experience walking on the AST. A future study to understand the difference between AST and FST walking on SSWS is warranted. Another limitation of this study includes participant recruitment sources. Many of the participants were individuals that participated in activities at a university-sponsored older adult gym or recruitment from word-of-mouth. These recruitment sources could have led the participant group to be biased towards high functioning and/or active older adults leading the participant group to not be a true representative of community-dwelling older adults. A limitation to the outdoor walking condition would be the seasonal environment in which participants walked in. We collected outdoor data during spring, summer, and fall seasons when outdoor temperatures were above 32° F and at least 24 h after precipitation. The colder temperatures may have caused the participants to walk with their eyes down to look potential perturbations, even when the sidewalk was cleared, or walk with a faster walking speed compared to the warmer weather. This may have an impact on how participants walked due to differences in visual flow between conditions.

## Conclusions and future directions

Older adults exhibit changes in peak ankle plantarflexion and peak knee flexion angles during outdoor walking compared to treadmill walking but not between treadmill controller types. The AST incorporates unconstrained walking speeds as a factor into AST controller through modulation of the push-off force, cadence, and step length. Even with this AST controller, we found no differences in the kinematics exhibited by older adults between both AST and FST walking. Further expansion on the changes in kinematic response exhibited between AST, FST, and outdoor walking is needed. Kinematics influence aspects of walking associated with dynamic stability such as margin of stability and center of mass Lyapunov exponent. Therefore, future studies are needed to understand how the dynamic stability of older adults’ changes in responses to AST, FST, and outdoor walking. These future studies will assist in understanding the extent to which the AST can be used to emulate outdoor walking for fall prevention interventions and falls related research.

## Methods

### Participant demographics

The general inclusion criterion was being between the ages of 60 and 85 years old. Exclusion criteria included a diagnosis of a neurological disorder (including, but not limited to, stroke, traumatic brain injury, Alzheimer’s, and dementia) and/or a diagnosis of osteoporosis. Eighteen older adults participated in the study. Two participants were excluded due to issues in equipment recordings. Two participants used the handrails during either or both treadmill conditions and were excluded from all data analyses due to handrail use affecting the outcome measures and would then be a confounding factor [22–24]. Fourteen older adult participants were included in the study analysis (sex: 12F/2M, age: 69.93 ± 4.53 years, mass: 74.7 ± 12.79 kg, height: 1.65 ± 0.1 m, BMI: 27.34 ± 4.14 kg/m^2^, M ± SD). All individuals signed an approved informed consent and the study was approved by the University of Nebraska Medical Center Institutional Review Board.

### Data collection

**Fig. 5:**
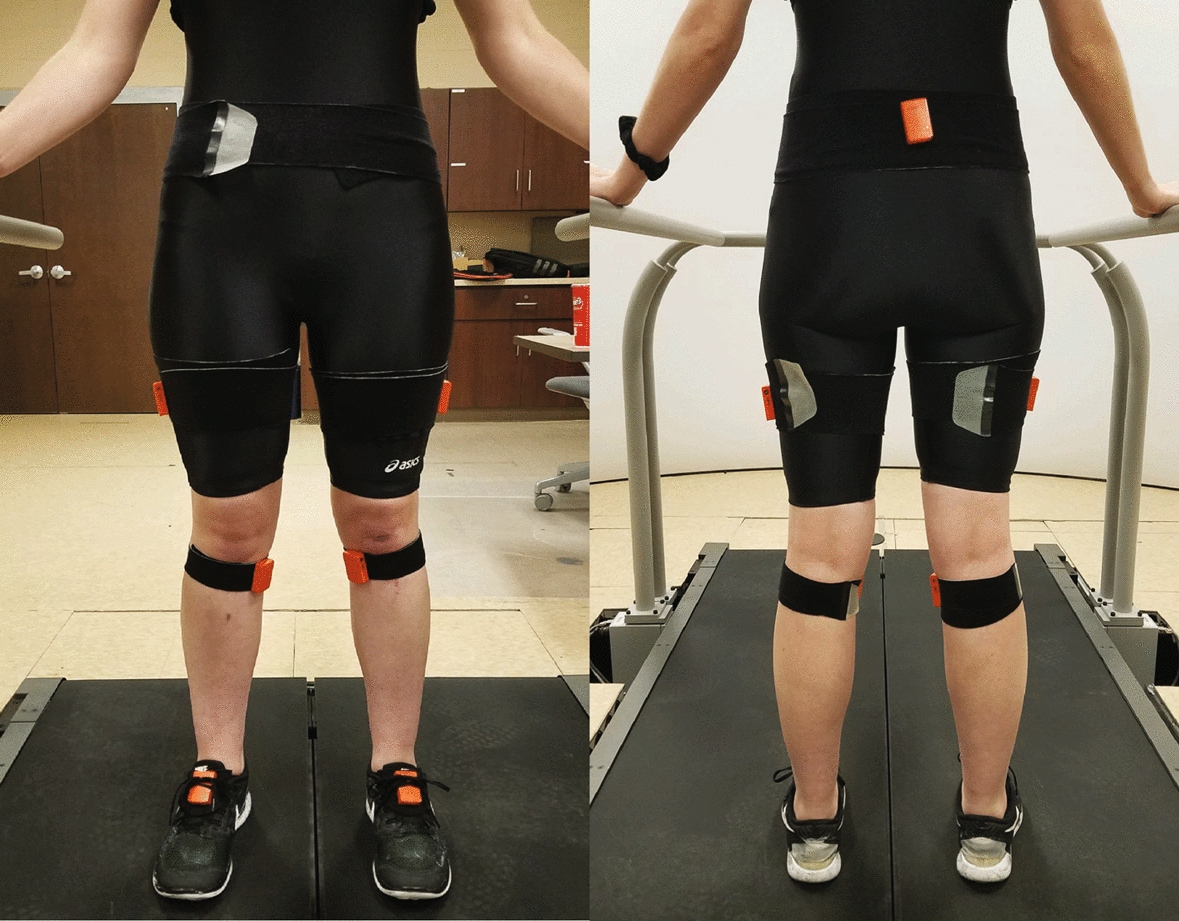
IMU-based motion capture sensor (orange) setup for data collection

Participants were asked to complete three walking conditions that included FST, AST, and outdoor walking conditions. The FST and AST conditions were randomized to eliminate any potential crossover or learning effects that may occur. Lower extremity kinematics were collected using an instrumented split-belt treadmill (Bertec, Columbus OH, USA) in conjunction with a wireless inertial measurement unit (IMU) motion capture system (Xsens Technologies B.V., Enschede, Netherlands). The IMU-based pelvis sensor was attached over the sacrum, the thigh sensors were attached bilaterally to the lateral mid-thigh, and the knee sensors were bilaterally attached medial of the tibial tuberosity, using elastic Velcro straps while the foot sensors were placed bilaterally on the dorsal foot under the tongue of participants’ shoes (Fig. 5). Participants were asked to not use the handrails during the treadmill conditions, but had the option to do so if they deemed necessary.

#### Self-selected walking speeds

For the Fixed Speed Treadmill condition, the SSWS was read directly from the treadmill controller interface. For the Adaptive Speed Treadmill condition, participants’ SSWS was calculated as the average speed from 30 s to 3 min of walking, using recording period data, from the real-time speed component of the Adaptive Speed Treadmill controller to ensure steady-state walking. The real-time speed component records the instantaneous treadmill belt speed for each frame. The real-time speed is calculated as:

$${\text{Real-time speed}}\, = \,{\text{realtimeSpeed}}\, + \,{\text{realtimeAccel }}*{\text{ acceldir }}*{\text{ deltaTime,}}$$

where realtimeSpeed is the current instantaneous treadmill belt speed. RealtimeAccel is the fixed treadmill belt acceleration set to 0.2 m/s^2^. The variable acceldir is the direction of acceleration where 1 indicates an increase in belt acceleration, −1 indicates a deceleration of the treadmill belts and 0 indicates no change in treadmill belt acceleration. The variable deltaTime is the frame rate (300 Hz) at which the real-time speed is recorded. For the outdoor walking condition, SSWS was calculated as the final distance, measured using a rolling measuring wheel, covered in 6 min over time then converted to m/s.

#### Fixed-speed treadmill condition

Participant’s SSWS was determined by initially setting the treadmill to 0.5 m/s then increasing the treadmill speed by increments of 0.1 m/s every 10 s until the participant verbally indicated they were walking at a comfortable speed “like walking in the park”. Participants were then asked to perform 3 min of FST walking at their SSWS with an IMU-based motion capture system.

#### Adaptive-speed treadmill condition

The AST condition used a treadmill controller that incorporates a set of inertial force, gait parameter, and position-based controllers that respond to the instantaneous anterior inertial force, step length, step time, and position of the participant on the treadmill to change the speed of the treadmill belts. Please refer to Ray et al. [12] for a more detailed controller description. Participants were verbally instructed to walk at a comfortable pace “like walking in the park.” A 2-min acclimation period was performed on the AST in order for the participants to become comfortable with walking on the AST, in accordance with previous studies [12, 25, 26]. Participants were allowed more acclimation time if they still did not feel comfortable at the end of the acclimation period. Data were not recorded during this acclimation period. Participants were then asked to complete 3 min of walking at their self-selected unconstrained walking speed with the IMU-based motion capture system.

#### Outdoor condition

**Fig. 6:**
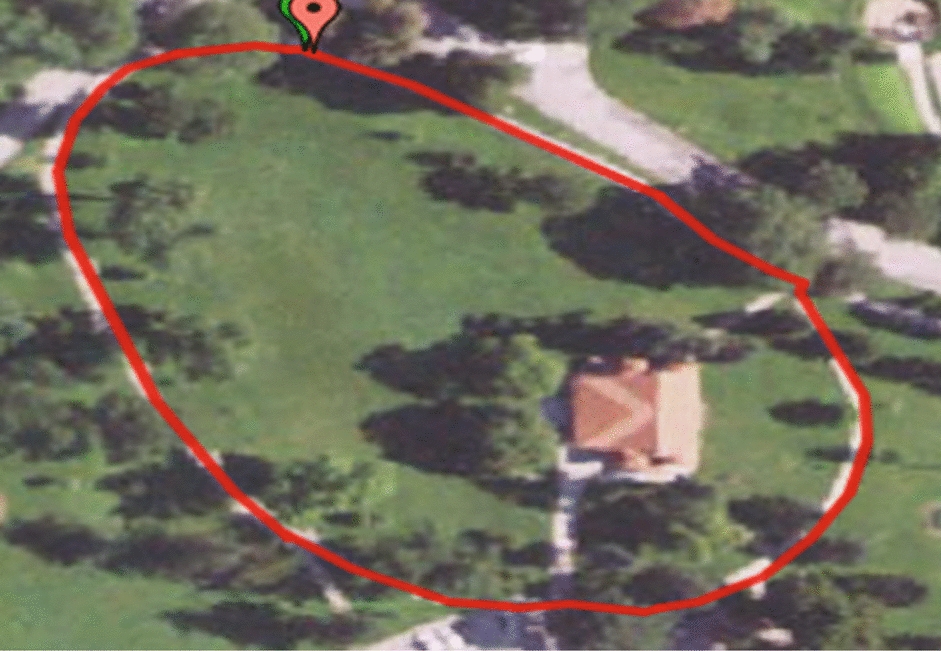
Outdoor circular walking route covering a distance of 380.78 m

Lower extremity accelerations and kinematics were collected using the IMU-based motion capture system during the outdoor walking condition. The IMU sensors were again placed at the posterior pelvis and bilaterally at the thigh, shank, and foot segments with elastic Velcro straps. The outdoor walking condition was conducted in a neighboring community park across the street from the data collection building following the route shown in Fig. 6. The route follows a circular geometry, similar to an indoor track, for a distance of 380.78 m. The participants walked along the route on sidewalk pavement for the entirety of the route. Participants were instructed to walk at a comfortable pace following the route for 6 min. Distances were recorded for every minute using a rolling measuring wheel. The sampling frequency of the IMU motion capture system was set to 60 Hz. The outdoor walking condition was performed when outdoor temperatures were above 32 °F and at minimum 24 h after precipitation. The paved sidewalk path was also cleared of obstacles prior to data collection.

### Data analysis

#### Self-selected walking speeds

For the FST condition, the SSWS was read directly from the treadmill controller interface. For the AST condition, each participant’s SSWS was calculated as the average speed from 30 s of the start of the AST to 3 min of recorded walking from the real-time speed component of the AST controller to ensure steady-state walking by excluding the acceleration period. For the outdoor walking condition, SSWS was calculated as the final distance covered in 6 min over time then converted to m/s. Data analysis was performed in both Visual 3D and MATLAB R2018a (MathWorks, Natick MA, USA).

#### Peak joint angles

The IMU-based motion capture data were used for analysis from the AST, FST, and outdoor walking conditions. The middle 3 min of the outdoor walking condition were used for analysis to compare to the 3 min of treadmill walking to ensure acceleration and deceleration periods were not included in data analysis. The IMU-based motion capture system uses proprietary algorithms to transform the sensor-based coordinate system to the global coordinate system in order to calculate joint angles [27]. Peak ankle plantarflexion peak knee flexion, and peak hip flexion were the variables of interest for this study. Ankle and knee joint angles were calculated and filtered using a low-pass Butterworth filter with a 6 Hz cut-off frequency. Peak ankle plantarflexion was calculated as the maximum plantarflexion angle between a foot segment and a referenced shank segment in the Cardan sequence of the capture space. Peak knee flexion was calculated as the maximum positive angle between the shank segment and a referenced thigh segment in the Cardan sequence of the laboratory. Peak hip flexion angle was calculated as the maximum negative angle between the thigh segment and the trunk in the Cardan sequence of the laboratory. All joint angles were analyzed in the sagittal plane with the peak of every stride used for analysis. The mean peaks were calculated from each stride across both left and right joints. The mean peak strides were then averaged between the left and right sides to get a single mean peak joint angle and standard deviation value for each participant for peak ankle plantarflexion, peak knee flexion, and peak hip flexion angles.

### Statistical analysis

A 3 × 1 repeated-measures ANOVA was performed comparing AST, FST, and outdoor conditions using SSWS data. Two 3 × 1 repeated-measures ANOVAs were also performed comparing AST, FST, and outdoor walking conditions using peak ankle plantarflexion angle data and peak knee flexion angle data, respectfully. Additionally, two repeated-measures analysis of covariance (ANCOVA) were performed comparing AST, FST, and outdoor walking conditions for peak ankle plantarflexion and peak knee flexion angles, controlling for SSWS in each condition. Partial Eta squared (*η*^2^_*p*_) was calculated as an indicator of effect size, with intervals defined as small (*η*^2^_*p*_ = 0.01), medium (*η*^2^_*p*_ = 0.06), and large (*η*^2^_*p*_ = 0.14) [28]. Post hoc Tukey t-tests were also performed to determine where the difference in walking conditions occurred. Due to failing the Shapiro–Wilk test for normality, peak hip flexion angles for the AST, FST, and outdoor conditions were compared using a Friedman’s test. Hedges g effect size with sample size bias correction was used to determine the strength of the post hoc t-test results. Effect size intervals are defined as small (*d* = 0.2), medium (*d* = 0.5), and large (*d* = 0.8) [28]. Statistical analyses were performed in SPSS (IBM, Armonk NY, USA) with statistical significance set to *α* ≤ 0.05.

## Data Availability

The data are available on request from the authors. The data that support the findings of this study are available from the corresponding author, Sheridan M Parker, upon reasonable request.

## Abbreviations

AST: Adaptive-speed treadmill
FST: Fixed-speed treadmill
SSWS: Self-selected walking speed
IMU: Inertial measurement unit
